# Complementary and Alternative Medicine for Autism – A Systematic Review

**DOI:** 10.1007/s10803-024-06449-5

**Published:** 2024-07-08

**Authors:** Monica Doherty, Kitty-Rose Foley, Janet Schloss

**Affiliations:** 1 Faculty of Health, National Centre for Naturopathic Medicine, Southern Cross University, 1 Military Road, Lismore, NSW 2480 Australia; 2 Faculty of Health, Southern Cross University, Gold Coast, Qld 4225 Australia

**Keywords:** Autism, Autistic, Complementary and alternative medicine, Dietary interventions, Nutraceuticals

## Abstract

Complementary and Alternative Medicine (CAM) is a therapeutic option currently used by autistic people with continued interest and uptake. There remains limited evidence regarding the efficacy of CAM use in autism. The aim of this systematic review is to comprehensively review published clinical trials to explore the efficacy of CAM in autism. A systematic literature review of available research published from June 2013 to March 2023 was conducted. Our literature search identified 1826 eligible citations, and duplications removed (n = 694) with 102 articles eligible for title/abstract screening. After full text review, 39 studies were included. The results of this systematic review identified that for autistic people, vitamin and mineral supplements may only be of benefit if there is a deficiency. The results also found that the main interventions used were dietary interventions and nutraceuticals, including targeted supplements, vitamins and minerals, omega 3 s and prebiotics, probiotics and digestive enzymes. The evidence does not support some of the most frequently utilised dietary interventions, such as a Gluten Free Casein Free (GFCF) diet, and the use of targeted nutraceutical supplements may be of benefit, but more conclusive research is still required to direct safe and effective treatment.

**Supplementary Information:**

The online version contains supplementary material available at 10.1007/s10803-024-06449-5.

## Introduction

Autism spectrum conditions (hereafter, autism) are characterized by challenges in social communication, interaction and flexibility, and the presence of repetitive behaviours or restricted interests (Bargiela et al., 2016). The ability to hyperfocus, attention to detail, a good memory, creativity and differences in sensory processing are also frequently described autistic traits (Russel et al., 2019). Data from the Centre for Disease Control (CDC) in the US detail autism prevalence rates of 1 in every 36 children, while reported prevalence rates in adults differ, with only 2.2% of adults formally diagnosed as autistic (Maenner et al., 2023). Historically, autism has been perceived as being more prevalent in males, however recent research has evolved to suggest that there may be differing phenotypic presentations of autism that do not align with previous conceptualisations, including a Female Autism Phenotype (Holtman et al., 2007). Male-to-female ratios for autism prevalence range in research from 10:1 to 2:1, with sex differences more pronounced when the reported autism is not associated with an intellectual disability (Loomes et al., 2017). Ratios also change markedly with age, and as females are stated to be able to articulate their inner experience more as they get older, with internalizing behaviours such as depression, anxiety and eating disorders more prevalent in autistic females (Hull et al., 2017).

At present, a diagnosis of autism is based on clinical observation and subjective measurement against internationally validated diagnostic tools, such as the *Diagnostic and Statistical Manual of Mental Disorders* (5th ed.; DSM–5; American Psychiatric Association, 2013) using criteria based on significant deficits in social communication and interaction, together with at least two types of restricted and repetitive interests and behaviours (RRBs). Identification of autism in adults and pathways to diagnosis in adulthood remain poorly understood (Happe et al., 2016). Currently, there are no definitive, diagnostic biomarkers available for autism, with initial studies in the 1990’s focusing on the identification of genetic variants associated with autism, however more recent research efforts have focused on investigating potential neurodevelopmental markers (Chaboun et al., 2022). Despite ongoing research efforts, the ‘cause’ of autism remains unknown, however several genetic and non-genetic risk factors have been identified that, alone or in combination, may be implicated in the development and subsequent diagnosis of autism for an individual, with the causation of autism remaining widely misunderstood (Sauer et al., 2021). Whilst there continues to be advances in relation to research on diagnosis, effective supports and treatment approaches for autistic people, most of this research remains based on autistic children (Howlin et al., 2012). The body of research on the experience of autistic adults remains limited, including research into the use of Complementary and Alternative Medicine (CAM) by autistic adults (Hofer et al., 2017). Pharmacologic interventions for the core presentations of autism remain limited, with Risperidone and Aripiprazole two medications approved for use in autism, with most prescribing continuing to be off label (Hellings, 2023). Several such medications that have shown efficacy for autistic people include anti-depressants and attention deficit hyperactivity disorder (ADHD) medications (Birch et al., 2017). Similar to calls for further research into CAM use for autistic people, there is also a demand for further controlled studies in the use of pharmacological interventions (Aishworiya et al., 2022).

In addition to what are described as the core presentations of autism, there are also commonly co-occurring conditions that are experienced by autistic people that can be both psychological and physiological in nature (Khachadourian et al., 2023). Epilepsy and co-occurring psychiatric conditions such as anxiety and mood disorders are more frequently experienced in autistic people than their neurotypical peers (Khachadourian et al., 2023). Autistic children and adults are at risk of having gastrointestinal issues such as constipation, diarrhea, and abdominal pain, with suggestions that the associated pathways in the gut–brain axis contribute to alterations in both behaviour and cognition (Wasilewskal & Klukowski, 2015). Sleep disorders are also commonly experienced by autistic people, which can have an impact on social interaction, day to day life and academic achievement, with evidence suggesting that increased oxidative stress may play a major role in the pathogenesis of these symptoms (Devnani & Hedge, 2015).

The use of Complementary and Alternative Medicine (CAM) in Australia remains high, with recent figures indicating that approximately 70% of the population are utilizing some form of CAM as part of their health care (Complementary Medicines Association Australia, 2021). Females have been found more likely than males to seek CAM support (Steel et al., 2018). CAM has also been found to be widely used by autistic people, with up to 95% reported to have utilized some form of CAM (Alwhabi & Sambamoorthi, 2016). CAM therapies are generally divided into five domains, including biologically based therapies, mind–body therapies, manipulative and body-based therapies, energy therapies and whole medical systems/alternative medical systems (Koithan, 2009). CAM treatments offer a variety of supportive actions for some of the physiological pathways related to autism, which may explain the consistent uptake of biologically based CAM therapies (Bent & Hedren, 2015). Despite their popularity however, the research is still lacking as to how CAM can effectively and safely support autistic people (Trudeau et al., 2019).

Brondino et al (2015) conducted a review into the use of CAM therapies for autism over the period of 2003–2013, with the aim of their systematic review being to provide a comprehensive overlook at the efficacy of CAM use in autism. The aim of this systematic review is to provide a comprehensive review of original research over the last 10 years (2013–2023) on the efficacy of CAM use in autism, conducted in the context of a rapidly evolving landscape of personalised CAM interventions and an increasing understanding of autistic phenotypical presentations and lived experience. The high usage of CAM in the autistic population indicates that evidence-based, effective, and safe CAM interventions for autistic children and adults demand urgent investigation (Castejon et al., 2021).

## Methods

This systematic review was registered with Prospero, prior to commencement, registration no: 459294. Research was conducted following the PRISMA guidelines.

The following databases were used for the search: PubMed, CINAHL Plus full text, EBSCO – Medline, Proquest and Cochrane. The search terms were as follows: Complementary medicine OR Naturopathy OR Nutrition OR Herbal Medicine OR Complementary and alternative medicine OR Alternative medicine OR Diet OR Lifestyle OR Supplements OR Nutraceutical OR Botanicals OR Plant OR Vitamins OR Dietary Intervention AND Autis* OR Autism OR ASD OR Autism Spectrum Disorder OR Autistic AND Randomised control trial OR Clinical trial or Trial AND (y_10[Filter])) Filters: in the last 10 years.

The search strategy was defined for research that was published between 2013 and 2023, was not limited to any specific country but was limited to articles only in English. The articles reviewed had no age limits and were not gender specific. All publications were reviewed for further relevant references. One researcher in the field was reached out to with a query on accessing a paper that there was difficulty in accessing. Two researchers (MD and JS) independently reviewed all information about the articles provided by the databases. Any discrepancies were solved by consensus.

The inclusion criteria were Randomized Controlled Trials (RCTs) and Clinical Trials from only original research, providing results on the effects of variety of biologically based CAM treatments for autistic people of all ages, including dietary interventions, vitamins and herbal medicines. Autism was defined according to internationally valid diagnostic criteria such as the DSM. Case reports and case series, commentaries, editorials, non-RCTs, retrospective studies, reviews, meta-analyses, or thesis, and on animals were excluded. Pharmaceutical medicines and non-biological CAM therapies including mind–body medicine, manipulative/body-based therapies, and energy therapies were not included in this review. All biologically based CAM treatments included in this review would fall under the SEC classification of a precursor safety event, which result in no detectable harm, based upon the relevant evidence (Throop & Stockmeier, 2011). The Johanna Briggs Institute Critical Evaluation tool for Systematic Reviews was applied to all selected articles.

This research was reviewed by a member of the autistic community prior to submission, and led by an adult who self-identifies as autistic and is a parent to a formally diagnosed autistic child.

## Results

Our literature search identified 1,826 clinical publications with 694 duplicates removed. After the title/abstract screening, 102 publications were obtained for full text review. After detailed evaluation 39 studies were included, with no studies from a hand search of references retrieved. It is noted that no studies on the use of herbal medicines for autism were included because of the search, screening and evaluation process. Supplementary Tables 1, 2, 3, 4, 5, included as supplementary material, detail the results of all studies included in this systematic review. Supplementary Figure 1 (Appendix 1) further explains the process of selecting articles (Page et al., 2021).

### See Supplementary Appendix 1—Figure 1. – PRISMA Flowchart Outlining the Methodological Process of Selection of Articles Included in the Systematic Review

In summary, 36 of the eligible studies focused on CAM use for autistic children, with only 3 eligible studies including both autistic children and adults as participants. Four of the eligible studies included only autistic male participants, and for most of the studies over 70% of the subjects were male. The majority of the research was based in the United States of America (USA) (n = 14), however this systematic review included research from 15 different countries around the world, offering a snapshot of research within the last decade relating to CAM use by autistic people. Nearly half of the studies included in this review focused on autistic traits as the primary outcome measure (n = 18), with 17 of the included studies reporting on both autistic traits and physiological outcome measures (e.g. anthropometric data, biochemistry, sleep and GIT measures) and 4 included studies focusing on physiological outcome measures as the primary outcome. Only two of the included studies, by Arnold et al (2019) and Nogay et al. (2021), utilized Quality of Life measures as a primary outcome measure, via the Paediatric Quality of Life (PedsQL) measure.

### Biologically Based CAM Treatments

The biologically based CAM treatments found in this review consist of dietary interventions and nutraceutical supplements. Of the studies reviewed, 12 focused on dietary interventions and 27 studies on nutraceutical intervention. For the purposes of this review, nutraceuticals will include targeted supplements, vitamins and minerals, omega 3 s and prebiotics, probiotics and digestive enzymes.

### Dietary Interventions

Dietary interventions are frequently trialled by autistic people (Onal et al., 2023). The Gluten Free Casein Free (GFCF) diet is an elimination diet that involves the removal of some proteins from the diet such as gluten and casein, which are found in wheat, barley, rye, triticale and cow’s milk containing products (Baspinar & Yardmici, 2020). Although the GFCF diet is a frequently administered dietary intervention, research indicates that there is insufficient evidence of its effectiveness (Millward et al., 2008). Studies on the GFCF diet in this review did not find statistically significant effects of a GFCF diet, with Hyman et al (2016) reporting no significant effect on measures of physiologic functioning, behaviour problems or autism traits for 12 weeks of both intervention and follow up. Included studies from Gonzalez-Domenech et al. (2019, 2020) also concluded that there were no significant behavioural changes assessed via the Autism Treatment Evaluation Checklist (ATEC), Emotional Regulation Checklist (ERC-III) and Aberrant Behaviour Checklist (ABC) outcome measures for the GFCF intervention group, and no association with urinary beta-casomorphin concentrations. No significant differences between groups for autistic symptoms, maladaptive behaviours, or intellectual abilities were reported by Piwowarczyk et al (2020) after a GF diet intervention, with a conclusion that a GF diet did not affect functioning of autistic children. Adams et al (2018) did report significant improvement in non-verbal intellectual ability, greater improvement in autistic presentation for both autistic children and adults following a Gluten Free, Casein Free, Soy Free (GFCFSF) diet, however it is noted that this study also included supplementation with a formula of vitamins, minerals, and targeted nutraceuticals (including EFAs, Epsom salt baths, carnitine, digestive enzymes), which may have contributed to the results.

Another increasingly popular biologically based CAM dietary intervention over the last decade is the Ketogenic Diet (Batch et al., 2020). Mu et al (2019) reported improvements in autistic behaviours via outcome measures including the Autism Diagnostic Observation Schedule (ADOS), Childhood Autism Rating Scale (CARS) and Gas Chromatology-Mass Spectrometry (GC–MS) following a 3-month Ketogenic Diet intervention. A comparison of a Ketogenic Diet intervention or a GF/CF diet intervention to placebo by El-Rashidy et al (2017) indicated that there were statistically significant improvements as a result of both the Ketogenic and GF/CF dietary interventions over a 6-month period in ATEC and CARS scores for autistic participants of the study, however results from the Ketogenic diet group scored higher in measures of cognition and sociability. In recent years, there has been a growing interest in the effects of Fermentable Oligosaccharides, Disaccharides, Monosaccharides, And Polyols (FODMAPs) on gastrointestinal health, that are included in fructose, lactose, fructans, galactans and sugar alcohols (Bellini et al., 2020). In the study by Nogay et al. (2021), there were no statistically significant differences in the ABC or the Paediatric Quality of Life Inventory (Peds QL) outcome measures for the low FODMAP diet group, however there was significant improvement in gastrointestinal symptoms for the Low FODMAP intervention group.

Camel milk is reported to be a useful CAM support for autistic children, as it may increase superoxide dismutase (SOD) and myeloperoxidase (MPO), and lower levels of oxidative stress, which is a suggested factor in the aetiology of autism (Shabo et al., 2005). Two studies were included in this review that were conducted by Al-Ayadhi et al. (2013) and (2015), reviewing the use of camel milk for autistic children, both reporting significant improvements in the CARS behavioural outcome measures for the camel milk (raw and boiled) intervention groups, as compared to placebo groups in trials of two weeks duration. Further outcome measures including the Social Responsiveness Scale (SRS), and ATEC and serum Thymus and Activation-Regulation Chemokine (TARC) also showed statistically significant improvements in the camel milk intervention groups for both the studies included by Al-Ayadhi et al. (2015) and the included study by Bashir et al. (2014), with significant improvement to antioxidant enzyme levels in the Al-Ayadhi et al (2013) study. A novel inclusion in the dietary intervention studies was a double-blind placebo-controlled trial conducted by Castejon et al (2021) with male autistic children only, on the use of Cysteine Rich Whey Protein (CRWP) powder. CRWP has been identified as a safe and effective precursor of the antioxidant glutathione, and the findings of the study from Castejon et al (2021) concluded that the CRWP intervention group demonstrated significant improvement in the Vineland Adaptive Behaviour Scale (VABS-II) behavioural assessment, glutathione levels, and some maladaptive /internalizing behaviours.

### Nutraceuticals

Nutraceuticals are a non-dietary form of biologically based CAM interventions widely used within the autistic population, which have received attention due to their potential therapeutic effects that may have less potential for adverse responses than pharmaceutical medicines (Nasri et al., 2014). L-Carnosine is an amino acid which may offer neuroprotective, antioxidant and anti-convulsive properties, which has been reported to be of benefit to autistic children (Abraham et al., 2021). Of the studies on L-Carnosine included in this review, Mehrazad- Saber et al. (2018) concluded that 500 mg of L-Carnosine significantly improved sleep duration, and reduced parasomnias and total sleep disorders, but did not have a significant effect on anthropometric indices such as height, weight and BMI or the Gilliam Autism Rating Scale 2 autism presentation outcome measures. Abraham et al (2020), however, concluded that there were no statistically significant differences in the L-Carnosine intervention group. The intervention with L-Carnosine did not improve total scores of the CARS2-ST or ATEC measures, nor make any improvement to Bedtime issues, Excessive daytime sleepiness, night Awakenings, Regularity and duration of sleep and Snoring (BEARS or the 6-item Gastrointestinal Severity Index (6-GSI) scores for autistic children from baseline to the end of the two-month intervention. The study by Rhagavan et al. (2022) included in this review, found that there was a decrease in the CARS outcome measure score for the beta glucans intervention group, and that plasma levels of alpha-synuclein were significantly higher for the beta glucan intervention group than for that of an L-carnosine intervention group. The results by Mousavinejada et al. (2018), found that CoQ10 supplementation in autistic children improved markers of oxidative stress, with a correlation between improved serum Malondialdehyde (MDA), Total Antioxidant Status (TAS) and antioxidant enzyme values and a decrease in the CARS outcome measure score.

Sulforaphane, a dietary isothiocyanate that is found in cruciferous vegetables, is a nutraceutical that has had an increasing research focus over the last decade regarding its benefits of use by autistic children and adults (Ou et al., 2022). Of the studies reviewed, Magner et al (2023) concluded that the ABC and SRS-2 outcome measures raw scores improved in both the sulforaphane intervention and control group, with these findings like the results of the ADOS-2 subscale scores. Zimmerman et al (2021) similarly concluded small, non-statistically significant improvements on the Ohio Autism Clinical Impressions Scale (OACIS) outcome measures, however there was no reported improvement in the ABC outcome measure after sulforaphane intervention over 36 weeks. In contrast, Singh et al (2014) reported statistically significant and clinically meaningful improvements in the ABC, SRS and CGI-I outcome measures for autistic male subjects aged 13–27 years in the sulforaphane intervention group over 18 weeks, with total scores on outcome measures increasing back to that of pre-intervention upon discontinuation of treatment. In addition, the SRS-2 non-randomised analysis for length of exposure to sulforaphane reported by Zimmerman et al (2021) confirmed significant improvement in the sulforaphane intervention group for autistic children.

### Omega 3

The omega-3 and omega-6 families are the two most common classes of Poly Unsaturated Fatty Acid (PUFA) compounds (Veselinovic et al., 2021). Mazahery et al (2019) reported improvements to SRS, balance and motion outcomes in autistic children after omega 3 supplementation for 12 months. Parellada et al. (2017) reported statistically significant improvements in social motivation, communication scores and erythrocyte membrane omega 3/6 ratio in autistic children after 8 weeks. Bent et al. (2019) reported a reduction in hyperactivity in autistic children after 3 months intervention. Doaei et al (2021) described significantly improved stereotypical behaviours, social communication and Gait Abnormality Rating Scale (GARS) outcome measure scores in autistic children as reported findings for the omega 3 intervention groups after 8 weeks. Conversely, there was no improvement found in autistic presentation in the Strengths and Difficulties Questionnaire (SDQ) nor a better anti-inflammatory or fatty acid state was found in the study conducted by de la Torre-Aguilar et al (2022). Voigt et al. (2014) found omega 3 DHA intervention did not improve the core presentations of autism on the Clinical Global Impression-Global Improvement (CGI-I) outcome measure, while Mankad et al (2015) concluded that there was no improvement with 1.5 mg omega 3, with the Behaviour Assessment System for Children (BASC-2) externalizing problem score worsening with omega 3 supplementation.

### Vitamins and Minerals

Vitamin and mineral supplements are widely used by autistic children and adults alike, with benefits reported including addressing nutrient deficiencies, treating metabolic issues and improving quality of life (Adams et al., 2022). Included in this systematic review are studies on folinic acid, vitamin B12, and vitamin D. Of the included studies on folinic acid, Renard et al (2020) supplemented with folinic acid in autistic children and found that the ADOS global outcome measure and social interaction and communication sub scores were significantly improved over a 12-week period. Frye et al. (2019) reported that the folinic acid group showed significant improvement in VABS, ABC, Autism Symptom Questionnaire (ASQ) and BASC outcome measures in verbal communication CELF-preschool-2, CELF-4 and the Preschool Language Scale-5 (PLS-5).

Vitamin B12 has been identified as a potential treatment for the impaired methylation and sulphation capacity and low glutathione redox capacity associated with autism (Rossignol & Frye, 2021). The included study by Hendren et al. (2016) found that the Vitamin B12 intervention group showed significantly improved CGI measures, however no improvement was shown in the ABC or SRS outcome measures, concluding that these results may be reflective of the methylation capacity of individual subjects. Feng et al (2017) concluded there was a significant reduction in CARS and ABC outcome measure scores and improvement in language subscale stores for the Vitamin D3 supplementation intervention group. Javadfar et al (2020) reported an improvement to not only serum 25 (OH)D levels in the Vitamin D intervention group, but also statistically significant improvements to the CARS and ATEC outcome measure scales. Mazahery et al (2019) reported that Vitamin D intervention, combined with Omega 3, conferred statistically significant improvements in outcome measures of social awareness, social and communicative function, taste and smell. Kerley et al., 2017, however, noted that whilst Vitamin D supplementation improved serum Vitamin D levels, the only outcome measure that indicated any significant improvement was the self-care subscale from the Developmental Disabilities Modification of Children’s Global Assessment Scale (DD-CGAS).

### Digestive Enzymes, Prebiotics and Probiotics

Digestive enzymes aid food absorption by breaking down large molecules of food into smaller molecules, allowing for easier absorption and digestion (Ianiro et al., 2016). Saad et al (2015) concluded that there were significant improvements in the digestive enzyme intervention group in the CARS and Global Behaviour Rating Scale (GBRS) outcome measures of emotional response, general impression, general behaviour, restricted, repetitive and stereotypic behaviours and reported gastrointestinal symptoms such as quality of stools, abdominal pain and food variety. Prebiotics are relatively recent inclusion as a CAM therapy option for use by autistic people, and their effects are not yet well documented in scientific literature (Song et al., 2022). The study conducted by Grimaldi et al (2018) on the use of prebiotics by autistic people, in addition to the study from Wang et al. (2020) that reviewed the impact of prebiotics and probiotics being taken together, detailed improvement in anti-social behaviour, beneficial bacteria levels and changes to faecal and urine metabolites in the Prebiotic intervention groups. An exclusion diet, in addition to prebiotic intervention also significantly lowered abdominal pain and improved bowel movements. Wang et al. (2020) found improvement in ATEC outcome measure scores, inhibition of harmful bacteria, promotion of growth of beneficial bacteria and an increase in beneficial Short Chain Fatty Acid (SCFA) concentration in the Prebiotic plus Probiotic intervention group.

Probiotics are live microorganisms that in appropriate doses may confer a health benefit on the host, also having a potentially regulatory effect on some of the co-occurring gastrointestinal symptoms experienced by autistic people (Feng et al., 2023). Several studies on the use of probiotics were included in this review, with mixed results, with the specificity of the probiotic strain applied an important factor. Arnold et al (2019) reported that all outcome measures improved from baseline, with the PedsQL measure correlating significantly with abundance of Lactobacillus and the Peds QL and Parent Rated Anxiety Scale (PRAS) ASD both showing improvements. It was also noted that there were significant improvements in GI complaints for the Probiotic intervention group compared to placebo. Sanctuary et al (2019) concluded that the SNAP-IV outcome measure and several elements improved significantly in the probiotic intervention group, and a reduction in the frequency of GI symptoms and reduced occurrence of specific aberrant behaviours occurred in the B.infantis Probiotic intervention group. Liu et al (2019) reported that oppositional and defiant behaviours measured by the ABC outcome measure, were ameliorated completely with the administration of the Lactobacillus Plantarum PS128 Probiotic. There was a statistically significant increase from baseline Vineland-3 Adaptive Behaviour Composite score, and a trend for increased social/geometric viewing ratio following SB-121 Probiotic intervention in the study by Schmitt et al (2023).

## Discussion

CAM is a common therapeutic option utilized by autistic people globally (Alam et al., 2022). Whilst historically CAM interventions for autism have had an erroneous focus on ‘curing’ autism, because of continued research, significant advocacy efforts and an increased understanding of the presentation and lived experience of being autistic, a shift in such narratives is required (Bent & Hedren, 2019). It is also noted in this systematic review, and potentially reflective of other research into CAM use by autistic people, that the research focus is on outcome measures based on the level/severity of autistic traits, and to a lesser extent, physiological and biochemical measures. What is clearly lacking in the studies included in this systematic review, is the prioritisation of Quality of Life as a primary outcome measure. How CAM support may serve to improve the quality of life of autistic people is an important consideration in the utilization of this modality. Such detail on quality of life may aid in providing important direction on which interventions, taken at what dosage, frequency and duration may make a meaningful difference to an autistic person’s experience of their life.

Whilst CAM offers the potential for meaningful improvements to the quality and experience of life for all autistic children and adults, the focus of both interventions and their associated research continues to be on ‘minimising’ or ‘eliminating’ the autism and it’s ‘symptoms’, rather than offering support in areas of significance that are identified by autistic people. More focused research on how CAM may support quality of life in autistic children and adults is urgently needed. An autism diagnosis is lifelong and research into safe and effective supports for autistic adults continues to be lacking, for both pharmacologic and CAM options (Christon et al., 2010). A focus on quality of life may be lacking in CAM research for autistic people because the research has historically been focused on children, with the presumption historically that autism is a condition of childhood (Alam et al., 2022). It is important to recognise that the support needs for any given autistic person will no doubt change and evolve over a life span (Onal et al., 2023).Focused research into CAM use by autistic adults who may be able to articulate their experience of a given CAM intervention and it’s impact on their quality of life would make a meaningful and significant contribution to the literature on CAM options for autistic people. Autistic adults may also be more likely to have agency in the CAM supports they choose to implement, as compared to children, and in participating in such research can play an important role in guiding future prescribing and research direction (Hofer et al., 2017).

There has been a noticeable shift over the last 10 years from CAM interventions that are particularly based on exclusion, such as the exclusion of certain foods, to an evolution to less invasive, more supportive treatments that are focused on the inclusion of functional foods and nutraceutical supplements as discussed in the results of this systematic review. This review has highlighted studies in which nutraceuticals that target some of the key physiological pathways and presentations that are associated with autism, such as oxidative stress, which are an increasingly key focus of research. An increasing shift to the use of nutraceuticals such as sulforaphane, coenzyme Q10, prebiotics and strain specific probiotics is noted, with no studies included in this review on previous ‘first line’ CAM supports for autism such as zinc, magnesium and Vitamin B6. This may reflect an evolution of CAM research shifting from general one size fits all ‘protocols’ for autistic people, to more targeted, personalised interventions (Onal et al., 2023).

There were no studies included in this review on Dimethylglycine (DMG), N-Acetyl Cysteine (NAC) or Palmitoylethanolamide (PEA), all novel nutraceutical approaches that currently have limited research to support their use by autistic people but warrant further review as potentially valid and safe CAM supports (Bent & Hedren, 2015; Colizzi et al., 2021; Dhanjal et al., 2022). The limited existing research on use of DMG by autistic people demonstrates improvements in verbal communication, social interaction, affection and minimisation of repetitive behaviours (Dhanjal et al., 2022). The action of DMG is said to have a protective effect from physical, environmental and metabolic stress, however further research is required to understand the mechanisms of action of DMG in the human body and its interaction with the pathways implicated in autism (Dhanjal et al., 2022). Initial research into the use of NAC by autistic children indicates that NAC may be an option to reduce irritability in autistic children, hypothesised due to its antioxidant action, without the observed side effects of the existing pharmaceutical medications (Bent & Hedren, 2015). PEA supplementation may modulate immune response, neuroinflammation, mitochondrial function, and microbiota activity, and may improve quality of life, including the experience of common co-occurring conditions experienced by autistic people (Colizzi et al., 2021).

The results of this systematic review confirm that for autistic people, vitamin and mineral supplements may only be of benefit if there is a deficiency. Moreover, the evidence does not support some of the most frequently utilised dietary interventions, such as a GFCF diet. The use of targeted nutraceutical supplements may be of benefit, however more conclusive research is still required to identify safe and effective treatments. The results of this systematic review also demonstrated the contradictory/inconsistent evidence regarding the effectiveness of CAM treatments for autism. For any given intervention, there may be reported variance in results, with the included study by Voigt et al. (2014) a given example, with no improvement reported in the primary outcome measure, yet differing/varying improvements in parent and teacher outcome measures. Whilst there continues to be an increasing number of well-designed studies reviewing the safety and effectiveness of an increasing variety of dietary and nutraceutical interventions, more rigorous research is required. Such research is essential to review the potential benefits and harms, safety, cost considerations and efficacy on reported core characteristics and co-occurring conditions offered to autistic adults via CAM support, with a goal to inform individual decision-making and future clinical guidelines.

Serious side effects or adverse events were found to be rare in the studies included in this review, with some mild, transient side effects, such as nausea, abdominal pain and skin rashes reported, sometimes contributing to discontinuation/dropout by participants. Families of autistic children are often worried about the potential side effects of pharmaceutical medications, which may include adverse metabolic and cardiovascular effects and sedation (Stoner, 2017). CAM therapies, which are more likely to be associated with milder side effects than pharmaceutical medications, are becoming an increasingly popular alternative, with safety considerations and side effect severity potentially having an important influence on CAM uptake and use (Akins et al., 2010). If there is a CAM option available that may offer appropriate support to an autistic person, with fewer side effects and less risk of serious adverse events, these too should also be deemed as valid options, with appropriate research available to support their use. It is important that future research into CAM use by autistic people prioritises not only the efficacy, but the safety and financial accessibility of CAM therapies (Shuai et al., 2020).

Limitations consistent in most of the studies reviewed included small sample size, limited study length/duration, issues with compliance, consistent dropout rates and heterogeneity of the age of participants (with a focus on children under age 12 particularly). Also noted as a limitation is that the literature search is over a year old before publication. Such limitations must be considered in the interpretation of whether results of a given study are significant and meaningful. Compliance is an important consideration to note in discussing the results of the included studies, as it is a potential barrier common to the implementation of dietary interventions, particularly dietary interventions over a long duration, and the effectiveness of an intervention will be directly influenced by the compliance and consistent uptake of an intervention (Semple et al., 2011). It is also important when analysing the efficacy and use of dietary interventions for autistic people, particularly those of dietary exclusion, to consider the potential risks associated with such interventions (Yu et al., 2022). Given that restricted eating/feeding is more frequently experienced by autistic people, and the increased incidence of disordered eating and eating disorders, further increasing restrictions to dietary intake could prove to be problematic (Kerr-Gaffrey et al., 2020).

Finally, it is of the upmost importance that CAM support for autistic children and adults reflect the needs and wants of the autistic community, including a prioritisation of the consideration of the influence of social determinants, cultural factors and intersectionality, particularly in relation to gender identity (Zuckerman et al., 2015). Such considerations are critical as they may have a significant impact on the perceptions, understanding of and beliefs around autism, engagement in the diagnosis process, and which supports and/or interventions are chosen, if at all (Davenport et al., 2018). The de-stigmatisation of the autistic neurotype, an understanding of the breadth and depth of the varying autistic phenotypical presentations and the understanding that narratives around the autistic experience should be led by autistic people, are key factors in the shifts that have occurred over the last decade in CAM treatment approaches. Best practice CAM supports autistic people and ensures that as a priority any treatment recommendations accommodate the varied experiences and preferences of autistic people and their families, who are increasingly being empowered to articulate and validate their experiences of both their inner and outer worlds (Manzini et al., 2021).

## Conclusion

The best evidence of CAM treatments for autism based on the current literature in this systematic review are those that target specific vitamin or mineral deficiencies, such as Vitamin D, Omega 3 and Vitamin B12. In addition, nutraceutical interventions that support biochemical and physiological pathways and functions in the aetiology of autism including strain specific probiotics/prebiotics, digestive enzymes, sulforaphane, CoQ10 and camel milk may be beneficial. Whilst lacking in the included reviews, it is suggested that quality of life measures be prioritised in future studies on CAM use by autistic people, and that any research conducted with and on the autistic population should be led by and reflect the identified needs and wants of the autistic community.

## Supplementary Information

Below is the link to the electronic supplementary material.

Supplementary file1 (DOCX 75 KB)
