# Supplementary material for: Complementary and Alternative Medicine for Autism – A Systematic Review
Source: J Autism Dev Disord. 2024 Jul 8;55(10):3689–99. doi: 10.1007/s10803-024-06449-5 (PMC12476390; doi:10.1007/s10803-024-06449-5)
Supplement: Supplementary file 1 — Supplementary file1 (DOCX 75 KB) [file 10803_2024_6449_MOESM1_ESM.docx]

**Appendix 1**

**FIGURE 1. – PRISMA flowchart outlining the methodological process of selection of articles included in the systematic review**

**Identification of studies via databases and registers**

Records removed *before screening*:

Duplicate records removed (n = 694)

Records marked as ineligible by automation tools (n = 0)

Records removed for other reasons (n = 0 )

Records identified from:

Ebsco Medline (n = 366)

Ebsco Cinhal (n = 140)

Pubmed (n = 673)

Cochrane (n = 326)

Proquest (n = 321)

Registers (n = 0)

**Identification**

Records screened

(n = 1082 )

Records excluded

(n = 1032 )

Reports sought for retrieval

(n = 50)

Reports not retrieved

(n = 0 )

**Screening**

Reports assessed for eligibility

(n = 50 )

Reports excluded:

Wrong type of study (n = 11)

Studies included in review

(n = 39)

Reports of included studies

(n = 0)

**Included**

**Appendix 2 – Supplementary Tables**

**Table 1 – Dietary Intervention**

| **Author/**  **Year** | **Type/Duration of Study** | **Sample Size** | **Type of intervention/Dose** | **Comparator/Dose** | **Outcome Measure** | **Findings** |
| --- | --- | --- | --- | --- | --- | --- |
| Castejon AM et al, 2021 | Double-blind, randomized, placebo-controlled trial  Duration:  90 days | n=46 autistic children  (34m/6F)  Dropout:  6  Aged:  3-5 years | Daily Cysteine Rich Whey Protein in powder form 0.5 g/kg for children < 20kg or a 10-g dose for those >20kg)  n=19 | Placebo (rice protein mimicking the protein load in the intervention group)  n=21 | Plasma glutathione  CARS  PLS-5  SCQ  CBC  VABS-II | Significant improvement in VABS-II  Glutathione levels and maladaptive/internalizing behaviour for intervention group |
| Hyman SL et al, 2016 | Double-blind, placebo- controlled challenge study  Duration:  12 weeks of diet, then a 12-week follow-up. | n=14  autistic children  (12M/2F)  Dropout:  8  Aged:  3–5 years | Dietary challenges were delivered via weekly snacks that contained gluten, casein, gluten and casein  n=14 | Placebo – no dietary challenges | BSC  Sleep diary  CARS  Ritvo Freeman Real Life Rating Scale | No statistically significant effects for intervention |
| González-Domenech PJ et al, 2019 | Crossover randomised clinical trial  Duration:  6 months | n=32 autistic children  (25M/3F)  Dropout:  4  Aged:  3-18 years | Patients followed a diet including gluten and casein daily for three months and a GFCF diet for another three months  n=16 | Patients followed a GFCF diet daily for three months and then a diet including gluten and casein for another three months  n=12 | ATEC  ERC-III  ABC  24 hr recall  urinary concentrations of Beta-casomorphins, Hemogram, Biochemistry, weight/height monitoring, survey on eating behaviour and gastrointestinal symptoms | No significant behavioural changes  No association with urinary beta-casomorphin concentrations |
| Mu et al, 2019 | Double-blind, Randomised Pilot study  Duration:  3 months | n=17  autistic children  (gender not reported)  Aged:  2-14 years  n=10  non-autistic children  Aged:  2-14 years | Modified Ketogenic Diet - gluten free daily, addition of MCT oil (20% energy requirement) net 20-25g carbohydrate for autistic children  n=17 | Modified Ketogenic Diet - gluten free daily, addition of MCT oil (20% energy requirement) net 20-25g carbohydrate for non-autistic children  n=10 | ADOS  CARS  GC-MS  H NMR Spectroscopy ICP-MS | Improvements in all measures for intervention group and alterations in mitochondria related metabolites and trace elements |
| Al-Ayadhi et al, 2015 | Double blind, randomised, clinical trial  Duration:  2 weeks | n=65 autistic children  (60M/5F)  Aged:  2-12 years, | 500ml pasteurized camel milk daily  n=25  500ml unpasteurized camel milk daily  n=22 | Cow’s milk placebo 500ml daily  n=18 | CARS  SRS  ATEC | Significant differences in all measures with Camel milk intervention group |
| Bashir et al, 2014 | Prospective, double-blind, placebo-controlled trial  Duration:  2 weeks | n=45 autistic children  (40M/5F)  Aged:  2 to 12 years | 500ml boiled Camel Milk daily  n=15  500ml raw Camel Milk daily  n=15 | Cow’s milk placebo 500ml daily  n=15 | CARS  TARC | Significant differences in all measures for intervention group |
| Adams et al, 2018 | Randomized, controlled, single-blind study  Duration:  12-months | n=67 autistic children and adults  (55M/  12F)  Dropout:  12  n=50  non-sibling, non-Autistic children and adults  (41M/9F)  Aged  3–58 years | Autistic subjects received special vitamin/  mineral supplement, and additional treatments were added sequentially, including essential fatty acids, Epsom salt baths, carnitine, digestive enzymes, and a healthy gluten-free, casein-free, soy-free (HGCSF) diet daily  n=28  Autistic subjects with no intervention  n=27 | Non-autistic subjects received a special vitamin/  mineral supplement, and additional treatments were added sequentially, including essential fatty acids, Epsom salt baths, carnitine, digestive enzymes, and a healthy gluten-free, casein-free, soy-free (HGCSF) diet daily  n=50 | RIAS  VABS-II  CARS-12  SAS-PRO  PDRBI  ATEC  ABC  SRS  SSP  PGI-2  6-GSI  Handgrip strength  CBC  Chempanel  BMI  PUFA  CRP  Carnitine | Significant improvement in nonverbal intellectual ability in intervention group based on a blinded clinical assessment. Improvement in autism measures, developmental age, EPA, DHA, carnitine, and vitamins A, B2, B5, B6, B12, folic acid, and Coenzyme Q10 in semi-blinded clinical assessment |
| El-Rashidy et al, 2017 | Single centre study  Duration:  6 months | n=45 autistic children  (33M/  12F)  Dropout:  5  Aged:  3–8 years | Ketogenic diet as modified Atkins diet (MAD) daily  n=10  Gluten free casein free (GFCF) diet daily  n=15 | Balanced nutrition daily  n=15 | All patients assessed by neurological examination, anthropometric measures, CARS  ATEC | Both intervention groups showed significant improvement in ATEC and CARS scores  Ketogenic intervention diet showed more improvements in cognition and sociability as compared to GFCF diet group |
| Nogay et al  (79)  2021 | Single-site, randomized-controlled trial,  Duration:  19 days total  (2 weeks dietary intervention) | n=15  autistic children  (gender not reported)  Aged:  6-17 years | Low FODMAP diet group daily  n=7 | Habitual diet daily  n=8 | ABC  Peds QL  BSC | No differences in behaviour measures  Significant improvement in some GI symptoms for intervention group |
| Piwowarczyk et al, 2020 | A Randomized, Controlled, Single‐Blinded Trial  Duration:  8 months | n=66  autistic children  (56M/  10F)  Dropout:  8  Aged:  3-6 years | Gluten Free Diet daily  n=30 | Gluten Diet group consumed at least one normal meal containing gluten per day  n=28 | ADOS  SA/RRB  SCQ  Leiter IQ  ASRS  VABS2 | No differences between groups |
| Gonzalez-Domenech et al, 2020 | Randomised Clinical Trial  Duration:  12 months | n=37  autistic children  (29M/8F)  Dropout:  8  Aged:  2-18 years | 6-month Gluten Free/Casein Free diet, then crossover to 6 months on a diet containing Gluten and Casein  n=15 | 6 months on a diet containing Gluten/  Casein then crossover to 6 months on a Gluten Free/Casein Free diet  n=14 | ATEC  ERC III  ABC  24 hr recall  Urinary Beta-casomorphin | No significant change in behaviour with GF/CF diet |
| Al-Ayadhi et al, 2013 | A Double-Blind Randomised Clinical Trial  Duration:  2 weeks | n=60  autistic children  (gender not reported)  Aged:  2-12 | 500ml raw camel milk daily  n=24  500ml boiled camel milk daily  n=25 | Placebo group  (500ml cow’s milk daily)  n=11 | CARS  Serum GSH  Serum SOD  Serum MPO | Significant improvement to all measures in camel milk intervention group |

**KEY:**

**CARS** – Childhood Autism Rating Scale, **PLS-5** – Preschool Language Scale Fifth Edition, **SCQ** - Social Communication Questionnaire, **CBC** – Child Behaviour Checklist, **VABS-II** – Vineland Adaptive Behaviour Scale, **BASC** – Behaviour Assessment System for Children, **ATEC** – Autism Treatment Evaluation Checklist, **ERC III**– Emotional Regulation Subscale, **ABC** – Aberrant Behaviour Checklist, **ADOS** – Autism Diagnostic Observation Schedule, **SRS** – Social Responsiveness Scale, **TARC** – serum Thymus Activation Regulated Chemokine, **RIAS** – Reynolds Intellectual Assessment Scales, **SAS** – Social Anxiety Scale, **PDRBI** – Pervasive Developmental Disorder Behaviour Inventory, **SSP** – Short Sensory Profile, **DGI -2 –** Dynamic Geometric Images (eye tracking), **CBC** – Complete Blood Count, **PUFA** – Polyunsaturated Fatty Acids, **BMI** – Body Mass Index, **CRP** – C-Reactive Protein, **Peds-QL** – Paediatric Quality of Life, **SA/RRB** – Social Affect and Restricted and Repetitive Behaviours, **Leiter IQ** – Leiter International Performance Scale, ASRS – Adult ADHD Self Report Scale, **GSH** – Glutathione, **SOD** – Superoxide Dismutase, **MPO** – Myeloperoxidase.

**Table 2 – Nutraceuticals**

| **Author/**  **Year** | **Type/Duration of Study** | **Sample Size** | **Type of Intervention/Dose** | **Comparator/Dose** | **Outcome Measure** | **Findings** |
| --- | --- | --- | --- | --- | --- | --- |
| Mehrazad –Saber et al, 2018 | Double-blind, randomized clinical trial  Duration:  2 months | n=50  autistic children  (31M/  12F)  Dropout:  7  Aged:  4-16 years | 500 mg of carnosine daily  n=21 | 500 mg of placebo daily  (cornstarch)  n=22 | CSHQ  GARS2 | Intervention significantly reduced sleep duration, parasomnias and total sleep disorders score, improving sleep |
| Magner et al, 2023 | Prospective double-blind placebo-controlled study  Duration:  36 weeks | n=40  autistic children  (24M/  4F)  Dropout:  12  Aged:  3-7 years | Weight-based dosing of sulforaphane  (50 μmol SFN) daily  n=15 | Weight based dosing of spinach puree powder placebo daily  n=13 | ADOS-2  SRS-2  ABC | Mean raw scores on ABC and SRS-2 improved in both groups  Changes in ADOS-2 subscale scores consistent in both groups |
| Singh et al, 2014 | Placebo-controlled, double-blind, randomized trial  Duration:  18 weeks plus 4 week follow up | n=44  autistic children and adults  (M only)  Dropout:  4  Aged:  13–27  years | Sulforaphane  (50–150 μmol) broccoli sprout extract daily  n=26 | Indistinguishable  Placebo daily  (microcrystalline cellulose)  n=14 | ABC  SRS  CGI-I | Statistically significant and clinically meaningful improvements in ABC, SRS and CGI-I for intervention group |
| Zimmerman et al, 2021 | Randomized parallel double‐blind placebo‐controlled clinical trial  Duration:  36 weeks | n=57  autistic children  (41M/  4F)  Dropout:  12  Autistic:  3–12 years | Sulforaphane daily (tablet containing broccoli seed and sprout extract 15 μmol SF and active myrosinase  Enzyme)  n=22 | Placebo daily  (Identical microcrystalline cellulose and colour)  n=23 | OACIS  SRS  ABC | Small, non-statistically significant effects on OACIS-I for intervention group  Significant improvement measured by the ABC but not the SRS-2.  Non-randomized analysis for length of exposure showed significant improvements on both the ABC and SRS-2 for the intervention group |
| Raghavan et al, 2022 | Prospective, open-label, pilot clinical trial comprising of two arms  Duration:  90 days | n=18  autistic children  (gender not reported)  Dropout:  5  Aged:  3-18 years | Conventional treatment of behavioural therapies and supplemented with the L-carnosine 500 mg daily  n=4 | Conventional treatment of behavioural therapies and supplemented with the Nichi Glucan 0.5 g b.i.d daily  n=9 | CARS  Plasma alpha-synuclein levels | Significant decrease in CARS score in the Nichi Glucan intervention and increase in plasma levels of alpha-synuclein |
| Mousaveinejada et al, 2018 | Randomized, parallel, placebo-controlled study  Duration:  100 days | n=90  autistic children  (66M/24F)  Dropout:  12  Aged:  3-12 years | CoQ10 at doses of 30 and 60 mg daily  n=52 | Placebo daily  (indistinguishable starch)  n=26 | CoQ10  MDA  TAS  CARS  Data on children's behaviour collected from parents and babysitters | Improved MDA, TAS and CARS in intervention group |
| Abraham et al, 2020 | Randomized controlled trial  Duration:  2 months | n=67  autistic children  (43M/20F)  Dropout:  4  Aged:  3-6 years | l-Carnosine, 10–15 mg/kg in 2 divided doses daily plus Standard care of  occupational and speech therapy  n=31 | Standard care of occupational and speech therapy  n=32 | CARS2-ST  ATEC  BEARS  6-GSI | No difference in intervention or control groups |

**KEY:**

**CSHQ** – Children’s Sleep Habits Questionnaire, **GARS-2**- Gilliam Autism Rating Scale, **ADOS 2** – Autism Diagnostic Observation Scale Second Edition, **SRS 2** – Social Responsiveness Scale Second Edition, **ABC** - Aberrant Behaviour Checklist, **CGI-I** – Clinical Global Improvement Scale, **OACIS-I** – Ohio Autism Clinical Impression Scale, **CARS** – Childhood Autism Rating Scale, **MDA** – serum Malondialdehyde, **TAS** – serum Total Antioxidant Status, **CARS** – Childhood Autism Rating Scale, **ATEC** – Autism Treatment Evaluation Checklist, **BEARS** – Bedtime issues, Excessive daytime sleepiness, night Awakenings, Regularity and duration of sleep, Snoring

**Table 3 – Omega 3**

| **Author/**  **Year** | **Type/**  **Duration of Study** | **Sample Size** | **Type of Intervention/**  **Dose** | **Comparator/Dose** | **Outcome Measure** | **Findings** |
| --- | --- | --- | --- | --- | --- | --- |
| Parellada et al, 2017 | A randomized, crossover, placebo-controlled study  Duration:  8 weeks | n=77  autistic children  (gender not reported)  Dropout:  9  Aged:  5-17 years | Omega 3 (962 mg) daily for children and 1155 mg daily for adolescents  n=33 | Placebo  (paraffin oil)  n=35 | Erythrocyte membrane Fatty Acid composition Serum TAS  SRS  CGIS | Significant improvement in erythrocyte membrane Omega 6/3, Social Motivation and Social Communication scores with intervention |
| de la Torre-Aguilar et al, 2022 | Double-blind, randomized placebo-controlled intervention  Duration:  6 months | n=54 Autistic children  (36M/13F)  Dropout:  5  n=59 non-Autistic children  Aged:  2-6 years | 19 children received DHA/EPA 800 mg/day of DHA and 25 mg/day EPA  n=24 | Placebo  (non-DHA/  EPA lipid)  n=25 | Plasma lipids  Cytokines  FA profiles in plasma and erythrocytes | No clinical improvement |
| Bent et al, 2014 | Randomised, controlled trial  Duration:  6 weeks | n=69  autistic children  (50M/  7F)  Dropout:  12  Aged:  5-8 years | 1.3 grams of omega-3 fatty acids daily  n=29 | Identical placebo  (safflower oil)  n=28 | ABC  Parent/  teacher rated changes in hyperactivity | Greater reduction in hyperactivity in intervention group, not significant |
| Voight et al, 2014 | Randomised, double blind control study  Duration:  6 months | n=48 autistic children  (40M/  8F)  Dropout:  14  Aged:  3 to 10 years | 200 mg DHA daily  n=19 | Placebo  (corn/  sunflower oil)  n=15 | Parents and investigator CGII  Parents only  CDI  ABC  Parents and Teachers - BASC | No improvement on the CGI-I.  Parents rating higher for social skills on the BASC for placebo  Teacher rating higher for functional communication on the BASC for the intervention group |
| Doaei et al,  2021 | Double-blind, randomized clinical trial  Duration:  8 weeks | n=54 Autistic children  (39/15)  Aged:  5-15 years | 1000 mg omega-3 daily  n=28 | 1000 mg medium chain triglyceride placebo daily  n=26 | GARS-2  FFQ | Significant improvement in stereotyped behaviours, social communication, and GARS score in intervention group  No significant change in social interaction subscale |
| Mankad et al, 2015 | Randomized, double blind placebo-controlled trial  Duration:  6 months | n=38 Autistic children  (28M/10F)  Dropout:  1  Aged:  2-5 years | Omega-3 fatty acid 1.5g daily  n=18 | Placebo  (indistinguishable olive oil/MCT)  n=19 | PDDBI (Autism composite score)  BASC-2  (externalizing problems score), CGI-I, VABS-II, and PLS-4 | Intervention significant group by week interaction on BASC-2 externalising problem score  No significant difference between groups in PDDBI, adaptive function or language scores. |

**KEY:**

**TAS** – Total Antioxidant Status, **SRS** – Social Responsiveness Scale, **CGI** -– Clinical Global Impression Scale, **CDI** – Children’s Depression Inventory, **ABC** – Aberrant Behaviour Checklist, **BASC** – Behaviour Assessment System Children, **GARS-2** – Gilliam Autism Rating Scale, **FFQ** – Food Frequency Questionnaire, **PDDBI** - Pervasive Developmental Disorder Behaviour Inventory, **VBAS** - Vineland Adaptive Behaviour Scale, **PLS** – Preschool Language Scale.

**Table 4 – Vitamins and Minerals**

| **Author/**  **Year** | **Type/**  **Duration of Study** | **Sample Size** | **Type of intervention** | **Comparator** | **Outcome Measure** | **Findings** |
| --- | --- | --- | --- | --- | --- | --- |
| Mazahery et al, 2019 | Randomised  Placebo Controlled Trial  Duration:  12 months | n=117  autistic children  (gender not reported)  Drop out  n=44  Aged:  2.5-8 years | VID  Vitamin D3  2000 IU/day  n=31  OM  OM3 DHA  722mg/day  n=29  VID/OM both):  Vit D3 2000 IU and OM3 DHA  722mg daily  n=28 | Placebo (no detail provided on contents, appearance the same as intervention)  n=29 | SRS  SPM | VID – significant improvements in SRS social communicative function, taste/smell  OM-improvements in total balance and motion  VID/OM – significant improvements in SRS |
| Feng et al, 2017 | Clinical Trial  Duration:  3 months | n=215  autistic children  (42F/  173M)  mean age  4.76 + 0.95y  Drop out:  n=178  n=285 non-autistic children  (60F/225M)  mean age  5.12 + 1.15y | Vitamin D intramuscularly  Administered once a month at a dose of 150000 IU, in total 3 injections by a nurse and 400IU Vitamin D3 orally administered daily  n=37 | Non-autistic children, no intervention described  n=285 | ABC  CARS  Serum 25(OH) Vitamin D levels | Significant reduction in CARS score (P<0.016) and  ABC scores (P<0.038) for the intervention group |
| Renard et al, 2020 | Randomised Single-Blind Placebo Controlled Trial  Duration:  12 weeks | n=19  autistic children (gender not reported)  Aged:  3-10 years | Two doses of 5mg folinoral (folic acid) daily  n=9 | Placebo (no detail provided)  n=10 | ADOS global (sub scores of communication and social interaction)  SRS | Global ADOS score, social interaction and communication sub scores significant improvement in folinic acid group |
| Hedren et al, 2016 | Randomised Placebo Controlled Trial  Duration:  8 weeks | n=57  autistic children  (45M/12F)  Dropout:  7  Aged:  3-7 years | Methylocobalamin  (Methyl B12) at a dosage of 75mcg/kg given through a subcutaneous injection every 3 days  n=27 | Placebo of saline given through a subcutaneous injection every 3 days  n=23 | CGI  ABC  SRS | CGI score significantly improved in the Methyl-B12 group |
| Frye et al, 2018 | Randomised Double-Blind Placebo Controlled Trial  Duration:  12 weeks | n=48  autistic children  (39M/9F)  Dropout:  5  Mean age:  7 years 4 months | Folinic acid (2mg per kg) daily, maximum 50mg per day  n=19 | Placebo (no detail provided)  n=24 | CELF preschool 2  CELF 4  PLS-5  OACIS  VABS  ABC  SRS  BASC  AIM &  ASQ (parent only measure) | Significant improvements in the VBS, ABC, ASQ and BASC in the folinic acid group |
| Kerley et al, 2017 | Randomised Double-Blind Placebo Controlled Trial  Duration:  20 weeks | n=42  autistic children  (33M/9F)  Dropout:  4  Mean age  7.1 years | 2000 IU Vitamin D  Daily  n=18 | Placebo (no detail provided)  n=20 | ABC  SRS  DD-CGAS  Vit D | Improvement to self-care subscale on  DD-GAS in intervention group and increase to serum Vit D levels |
| Javadfar et al, 2020 | Parallel Randomised Double-Blind Placebo Controlled Trial  Duration:  15 weeks | n=52  autistic children  (36/7)  Dropout:  9  Aged:  3-12 years | Vitamin D drops  300 IU/kg up to a maximum of 6000 IU daily  n=22 | Placebo  (no detail provided)  n=21 | Serum 25-hydroxy-vitamin (OH)D  IL-6  Serotonin  CARS  ATEC  ABC | Significant improvement to Serum 25 (OH)D, CARS. ATEC |

**KEY:**

**TNF** – Tumour Necrosis Factor (serum), **ATEC** – Autism Treatment Evaluation Checklist, **SRS** – Social Responsiveness Scale, **SPM** – Sensory Processing Measure, **ABC** – Aberrant Behaviour Checklist, **CARS** – Childhood Autism Rating Scale, ADOS – Autism Diagnostic Observation Schedule, **CGI** – Clinical Global Impression Scale, **CELF** – Clinical Evaluation of Language Fundamentals, **PLS** – Preschool Language Scale, **OACIS** – Ohio Clinical Impressions Scale, **VBAS** - Vineland Adaptive Behaviour Scale, **BASC** – Behaviour Assessment System Children, **AIM** – Autism Impact Measure, **ASQ** - Autism Spectrum Quotient, **DD-GAS** -Developmental Disabilities Modification of Children’s Global Assessment Scale.

**Table 5 – Digestive enzymes, prebiotics and probiotics**

| **Author/**  **Year** | **Type/**  **Duration of Study** | **Sample Size** | **Type of intervention/**  **Dose** | **Comparator/Dose** | **Outcome Measure** | **Findings** |
| --- | --- | --- | --- | --- | --- | --- |
| Saad et al, 2015 | Double-blind, randomized clinical trial  Duration  3 months | n=101 autistic children  (82M/  19F)  Dropout:  9  Aged:  3 to 9 years | 15ml digestive enzymes in syrup daily  (5ml at the start of each meal)  n=47 | 15ml placebo daily  (5ml at the start of each meal of indistinguishable  sucralose syrup)  n=45 | CARS  GBRS | Intervention group had significant improvement in emotional response, general impression autistic score, general behaviour and gastrointestinal symptoms, restricted repetitive behaviours and stereotypic behaviours. |
| Wang et al, 2020 | Randomised controlled trial  Duration  1^st^ stage  12 months,  2^nd^ stage 30,60 and 108 days | n=26  autistic children  Mean age: 4.3 years  (24M/  2F)  n=24  non-autistic children Mean age: 4.5 years | Dietary intervention for 12 months then  Probiotic + fructo-oligosaccharide (FOS)  n=16 | Placebo group  (indistinguishable maltodextrin)  n=10 | ATEC  6-GI severity index  16s rRNA gene sequencing and analysis  UHPLC-MS/MS  Zonulin (ELISA) | Probiotic + FOS intervention significant improvement on questionnaires, harmful bacteria inhibit growth and beneficial bacteria promote growth and SCFA |
| Grimaldi et al,  2018 | Randomiseddouble-blind, placebo-controlled study  Duration:  10 weeks | n=41 autistic children  (31M/  10F)  Dropout:  11  Aged:  4–11 years | Exclusion or normal diet  Prebiotic B-GOS® mixture daily (Bimuno®; 1.8 g: 80% GOS content)  n=12 | Exclusion or normal diet  Placebo daily (Maltodextrin GLUCIDEX®; 1.8 g)  n=18 | Food diary DietPlan7  BSC  ATEC  SCAS-P  5-day sleep diaries  Faecal and urine samples | Exclusion diet significantly lower abdominal pain and bowel movement, Bifidobacterium spp. and Veillonellaceae family, higher Faecalibacterium prausnitzii  B- GOS® intervention, significant improvements in anti-social behaviour, increase of Lachnospiraceae family, and changes in faecal and urine metabolites |
| Arnold et al, 2018 | Randomized crossover feasibility pilot trial  Duration:  19 weeks | n=13  autistic children  (6M/  4F)  Dropout:  3  Aged:  3-12 years | 8 weeks on daily VISBIOME (dose), 3-week washout then daily placebo separated by a 3-week washout  n=6 | 8 weeks on Placebo daily then 3-week washout and 8 weeks of daily VISBIOME (dose)  n=4 | PedsQL GI module  Gut microbiota analysis  PRAS-ASD, Parent-selected target symptoms | Each outcome improved from baseline, intervention group more than placebo  PedsQL correlated significantly with abundance of Lactobacillus without discernible changes to microbiota composition/diversity  Significant improvement in GI complaints for intervention group |
| Liu et al, 2019 | Randomized, double-blind, placebo-controlled study  Duration:  4 weeks | n=80 autistic children  (M only)  Dropout:  9  Aged:  7-15 years | Lactobacillus plantarum PS128 capsule containing 3 × 1010 CFU with microcrystalline cellulose as the carrier daily  n=36 | Placebo capsules containing microcrystalline cellulose only daily  n=35 | ABC-T  SRS  CBCL  SNAP-IV  CGI-I | PS128 ameliorated opposition/defiance behaviours  Total score of SNAP-IV for younger children (aged 7−12) improved significantly  Several measures notably improved in the intervention group |
| Sanctuary et al, 2019 | Randomized, double blind, controlled trial  Duration:  12 weeks | n=11 autistic children  with GIT comorbidities  (7M/1F)  Dropout:  3  Aged:  2-11 years | Crossover study combination treatment Bovine Colostrum Powder (BCP) + *B*. *infantis* vs. daily for 5 weeks followed by a two-week washout period, and 5 weeks of prebiotic only supplementation daily  n=4 | BCP alone daily  n=4 | GIH  QPGS-RIII  ABC  RBS-R  ABAS-II  BSC  Faecal DNA  Microbiota analysis – QIIME  Blood  Urinary and Faecal samples | Reduction in frequency of certain GI symptoms and particular aberrant behaviours. |
| Schmitt et al, 2023 | Placebo‐controlled, double‐blind, crossover study  Duration:  28 days | n=15  autistic children and adults  (M only)  Aged:  15–45 years | Oral SB‐121 probiotic formulation daily, a combination of L. reuteri, Sephadex® (dextran microparticles)  n=7 | Maltose placebo daily  n=8 | VABS-III  Social preference measured with eye tracking | Statistically significant increase from baseline in the Vineland‐3 Adaptive Behaviour Composite score in intervention and trend for increased social/ geometric viewing ratio |

**KEY:**

**CARS** – Childhood Autism Rating Scale, **GBRS** – Gifted Behaviour Rating Scale, **ATEC** – Autism Treatment Evaluation Checklist, **BSC** – Bristol Stool Chart, **SCAS-P**- Spence Children Anxiety Scale (Parent), **PEDS-QL** – Paediatric Quality of Life, **PRAS-ASD** – Parent Rated Anxiety Scale for Autism Spectrum Disorder, **ABC-T –** Aberrant Behaviour Checklist – Temper, **SRS** – Social Responsiveness Scale, **CBCL** – Child Behaviour Check List, **SNAP-IV –** Swanson, Nolan and Pelham Rating Scale, **CGI-I –** Clinical Global Impression Scale, **GIH** – Growth Hormone Inhibiting Hormone, **QPGS-RIII** – Questions on Paediatric Gastrointestinal Symptoms Rome 3, **ABC** – Aberrant Behaviour Checklist, **RBS-R** – Repetitive Behaviour Scale – Revised, **ABAS-III -** Adaptive Behaviour Assessment System Third Edition, **BSC** – Bristol Stool Chart, **VABS-III** – Vineland Adaptive Behaviour Scale.
